# An increase of CD8^+^ T cell infiltration following recurrence is a good prognosticator in HNSCC

**DOI:** 10.1038/s41598-020-77036-8

**Published:** 2020-11-18

**Authors:** Yoon Kyoung So, Sun-Ju Byeon, Bo Mi Ku, Young Hyeh Ko, Myung-Ju Ahn, Young-Ik Son, Man Ki Chung

**Affiliations:** 1 Department of Otorhinolaryngology-Head and Neck Surgery, Ilsan Paik Hospital, Inje University College of Medicine, Goyang-Si, Korea; 2 Department of Pathology, Hallym University Dongtan Sacred Heart Hospital, Dongtan-Si, Korea; 3 Research Institute for Future Medicine, Samsung Medical Center, Sungkyunkwan University School of Medicine, Seoul, Korea; 4 Department of Pathology, Samsung Medical Center, Sungkyunkwan University School of Medicine, Seoul, Korea; 5 Department of Medicine, Samsung Medical Center, Sungkyunkwan University School of Medicine, Seoul, Korea; 6 Department of Otorhinolaryngology-Head and Neck Surgery, Samsung Medical Center, Sungkyunkwan University School of Medicine, 81 Irwon-ro, Gangnam-gu, Seoul, 06351 Korea

**Keywords:** Cancer microenvironment, Head and neck cancer

## Abstract

Programmed death-ligand 1 (PD-L1) expression and CD8-positive tumor-infiltrating lymphocyte (CD8^+^ TIL) infiltration are essential biomarkers for immune checkpoint inhibitor therapy. The objective of this study was to compare the expression of those biomarkers between initial and recurrent HNSCCs using paired analysis. Prognostic significance of those immunological changes was also investigated. Forty-two consecutive patients with locally recurrent HNSCCs were included. Immunohistochemical staining of CD8 and PD-L1 was done for both initial and recurrent tumor specimens. The IHC findings were verified with mRNA expression profiling. Also, the prognostic impact was analyzed based on overall survival (OS). Recurrent-to-initial (R/I) ratios of CD8^+^ TILs and PD-L1 were widely variable. CD8^+^ TIL density and PD-L1 expression decreased in 59.5% and 69% of patients, respectively (R/I ratio < 1). The R/I ratio of CD8A mRNA was significantly higher in patients with a CD8 R/I ratio > 1 (1.7 ± 1.5 vs. 0.6 ± 0.6, *p* = 0.042). CD8 R/I ratio (> 1) was a good prognosticator for OS (HR 0.293, 95% CI 0.091–0.945, *p* = 0.040). CD8^+^ TIL infiltration and PD-L1 expression changed variably following local recurrence of HNSCC. The increase of CD8^+^ TILs at recurrence was an excellent independent prognosticator.

## Introduction

Head and neck squamous cell carcinoma (HNSCC), the sixth most common malignancy, has more than 600,000 newly diagnosed cases annually worldwide^1,2^. Patients with HNSCCs often present with advanced stages, and more than half of these patients experience recurrences after initial treatment^3^. As a therapeutic strategy for recurrent cancers, cancer immunotherapy has recently shown promising outcomes in many cancer types, including HNSCC^4^.

It has been reported immunotherapeutic agents that target interactions between programmed cell death protein-1 (PD-1) and programmed death-ligand 1 (PD-L1) axis have overall response rates of 13.3–17.7% in recurrent and/or metastatic HNSCCs with noticeable improvements in overall survival (OS)^5–7^. It is essential to select appropriate patients using reliable biomarkers to ensure the treatment success of anti-PD1/PD-L1 immunotherapy. PD-L1 expression and CD8-positive tumor-infiltrating lymphocyte (CD8^+^ TIL) infiltration within the tumor microenvironment have been reported to be the two most promising predictors of treatment response to anti-PD1/PD-L1 immunotherapy and overall prognosis^8–12^.

Previous reports have shown that the expression of PD-L1 could change during radiotherapy or chemotherapy. Also, it is known that PD-L1 expression and CD8^+^ TIL infiltration can change during the clinical course of HNSCC from the initial diagnosis to recurrence^13–15^. However, few studies have been conducted on the pattern of onco-immunologic biomarkers in recurrent tumors, given that these changes in immunologic properties could affect oncological outcomes and responses to cancer immunotherapy.

Thus, this study's objective was to determine differences of CD8^+^ TIL infiltration and PD-L1^+^ expression between initial and recurrent HNSCCs by paired analysis in each patient. The expression of each biomarker in a tumor specimen was measured by immunohistochemical (IHC) staining, and related mRNA expression pattern was correlated. We evaluated whether such changes in the expression of those biomarkers have any impact on the oncological outcomes.

## Materials and methods

### Patients

The Institutional Review Board approved this study of Samsung Medical Center (SMC IRB file no. 2015-11-073) and was conducted following the Helsinki Declaration. Of patients who had undergone surgery for initial HNSCCs from March 2000 to December 2015, only the patients who had local or locoregional recurrences were included in this study. Of them, patients for whom pathological specimens of initial or recurrent tumors were unavailable were excluded. Patients with initial treatment failure or remnant disease and those with only regional or distant recurrence after surgery-based initial treatment for HNSCC were also excluded. Finally, 42 consecutive patients were included for further analysis. Their pathological specimens of initial and recurrent tumors were obtained for paired analysis. Informed consent was waived by the Institutional Review Board of Samsung Medical Center because this study was conducted retrospectively, and the follow-up had ended up for most patients.

Clinicopathological profiles of 42 patients analyzed in this study are presented in Table 1. The mean age of these patients was 59.5 years. There were 32 males and 10 females. The most common tumor site was the oral cavity (21 cases, 50.0%), followed by the larynx (15 cases, 35.7%). At the initial presentation, 73.8% of patients showed early T classification (T1: 21 cases, T2: 10 cases), and 59.5% had no lymphatic metastasis at initial treatment (N0: 25 cases). Among all patients, 28.6% (12/42) of them were treated only with surgery for their initial tumors, whereas 71.4% (30/42) received adjuvant radiotherapy (RT) after the initial surgery. The mean follow-up period was 61.0 ± 48.9 months (range, 5–212 months).

**Table 1:** Study patients (N = 42).

Characteristics		Values
Age (year)		59.5 ± 14.8^a^
Gender (male/female)		32/10
T classification^†^	T1	21 (50.0%)
	T2	10 (23.8%)
	T3	4 (9.5%)
	T4	7 (16.7%)
N classification	N0	25 (59.5%)
	N1	5 (11.9%)
	N2	12 (28.6%)
	N3	0 (0%)
M classification	M0	42 (100%)
Stage^†^	1	18 (42.9%)
	2	1 (2.4%)
	3	8 (19.0%)
	4	15 (35.7%)
Tumor sites	Oral cavity	21 (50.0%)
	Larynx	15 (35.7%)
	Oropharynx	2 (4.8%)
	Hypopharynx	2 (4.8%)
	PNS	2 (4.8%)
Adjuvant RT	Not performed	12 (28.6%)
	Performed	30 (71.4%)
Adjuvant CT	Not performed	38 (90.5%)
	Performed	4 (9.5%)
Follow-up period (m)		61.0 ± 48.9^a^

*PNS* paranasal sinus, *RT* radiation therapy, *CT* chemotherapy.

^a^Mean ± standard deviation, ^†^TNM classification & staging according to AJCC 7th edition.

### Paired analysis of immune-oncologic biomarkers in initial and recurrent tumors

Formalin-fixed paraffin-embedded (FFPE) tumor samples of initial and recurrent tumors were obtained from archives. Unstained slides were produced for all 84 FFPE tumor samples. IHC staining was performed for CD8 and PD-L1 using FFPE slides. IHC staining of CD8 (1:200, clone 4B11, Leica Biosystems, Nussloch, German) was done using an automated immunostainer (BOND-MAX Automated IHC/ISH Stainer, Leica Biosystems). PD-L1 IHC staining (1:25, clone SP142; Spring Bioscience, Pleasanton, CA, USA) was performed with another automated immunostainer (Benchmark XT, Ventana, Tucson, AZ, USA). Each IHC staining's signals were visualized using an Optiview DAB IHC detection kit (Ventana, Catalog number 760-700) and an Optiview Amplification kit (Ventana, Catalog number 860-099).

**Figure 1:**
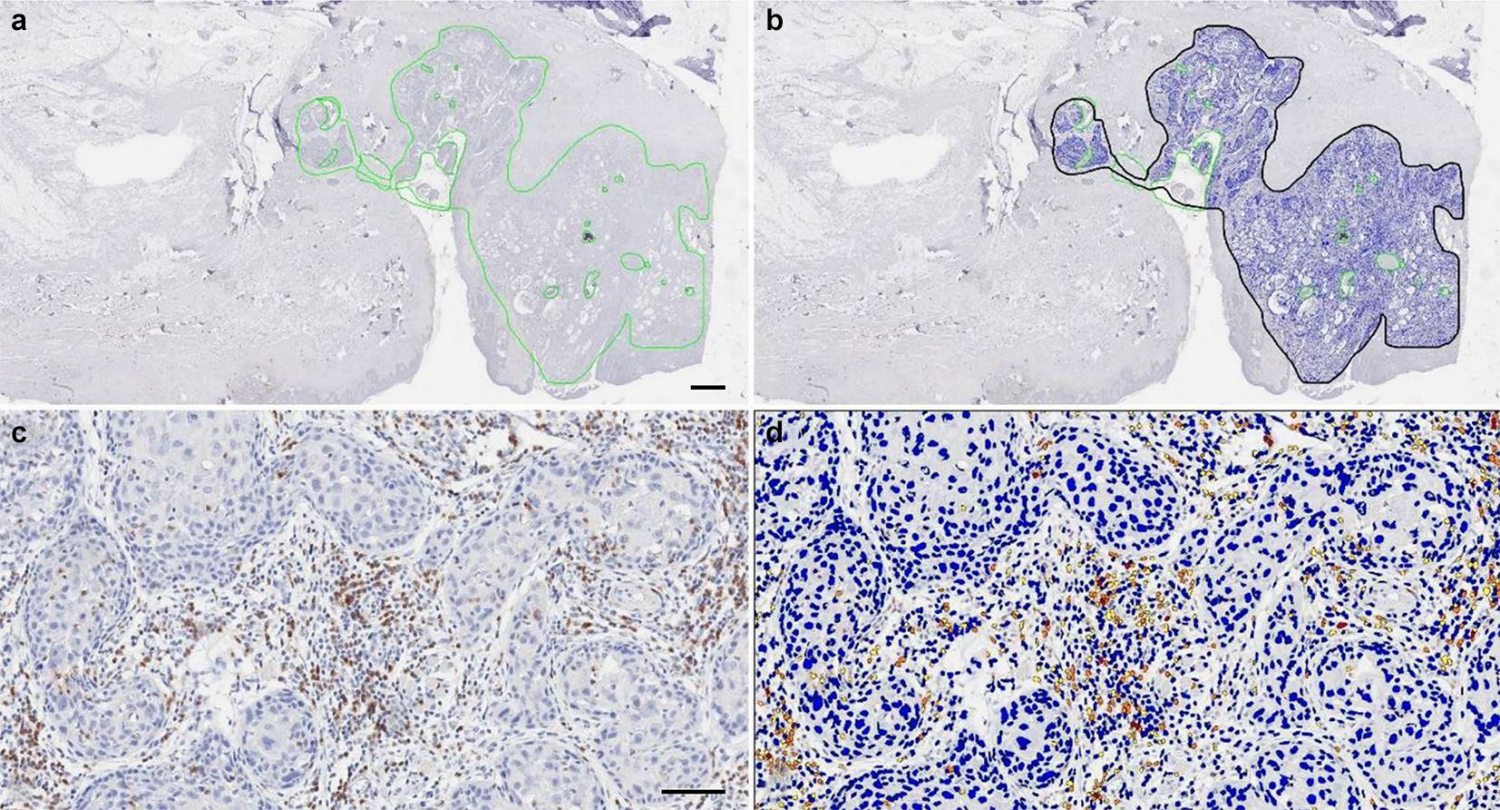
Immunohistochemical staining (IHC) and measurement of CD8^+^ tumor-infiltrating lymphocytes (TILs). (**a**) Low magnification image with an indication of tumor area by a solid green line. Areas of cystic space, non-specific stain, and dust were excluded from analysis (scale bar = 1 mm). (**b**) Converted image of 1A for the analysis scanned by Aperio (Leica Biosystems). (**c**) Representative image of high magnification. Many lymphocytes within the tumor area were stained with IHC staining for CD8 (scale bar = 50 μm). (**d**) Representative Aperio image of 1C. Lymphocytes are marked with blue (negative), yellow (1 +), orange (2 +), or brown (3 +) depending on staining intensity.

For further morphometric analysis, IHC slides for CD8 and PD-L1 were scanned under high-power magnification (× 20) using an Aperio AT2 (Leica Biosystems). A pathologist marked the entire tumor region that contained the central tumor and the invasive margin on a representative slide without clinical information (Fig. 1). The number of CD8^+^ TILs was measured using an Aperio Scanscope Nuclear version 9 algorithm as previously described^16^. The density of CD8^+^ TILs was obtained by dividing the number of CD8^+^ TILs by the marked region area. PD-L1^+^ cells were measured using an Aperio Scanscope Positive Count Pixel version 9 algorithm as previously described^17^. All PD-L1^+^ cells, whether tumor cells or TILs were counted, and the density of PD-L1^+^ cells was determined by dividing the number of positive pixels by the marked region area. For each biomarker, the densities of CD8^+^ TILs and PD-L1^+^ cells in the recurrent tumor (R) was divided by their densities in the initial tumor (I) to obtain an R/I ratio.

### mRNA expression profiling of immune-related genes

After excluding specimens with too low mRNA density (less than 100 ng), 33 cases (66 specimens, 33 initial and 33 recurrent tumors) were included in mRNA expression analysis. Total RNAs were extracted from FFPE tissue sections using an RNeasy FFPE Kit (Qiagen). RNA yield and purity were assessed using a DS11 Spectrophotometer (Denovix Inc, DE, USA), and RNA quality was checked using a Fragment Analyzer (Advanced Analytical Technologies, IA, USA).

Digital multiplexed NanoString nCounter human mRNA expression assay was performed with > 300 ng total RNA isolated from FFPE tissue and Human Pancancer Immune Profiling Panel Kit (NanoString Technologies, Seattle, WA, USA). Human Pancancer Immune Profiling Panel Kit included 730 immune-related genes and 40 housekeeping genes with positive and negative controls. Nanostring assay was conducted according to the manufacturer's protocols, described previously^18^. mRNA profiling data were normalized using 40 housekeeping genes, including AGK, G6PD, and TUBB. The normalized value of each gene in recurrent tumor tissue was divided by the value in the corresponding initial tumor tissue and expressed as R/I ratio of each gene.

### Statistical analysis

An independent *t*-test was used to compare the continuous variables of the two groups. Overall survival (OS) was assessed using Kaplan–Meier estimates. A log-rank test was used to assess the equality of survival function between different groups. The Cox proportional hazards model with a 95% confidence interval was used for multivariate analysis to assess significant survival factors. SPSS software for Windows, version 17.0 (SPSS, Inc., Chicago, IL, USA) was used for all statistical analyses. All tests were two-sided, and a *p*-value of less than 0.05 was considered statistically significant for all analyses.

## Results

In this study, the mean period to the first local or locoregional recurrence was 20.5 ± 3.3 months after initial treatment. Of clinical and immunological characteristics of initial tumors, initial T classification and initial N classification were significantly related to recurrence. Patients with T1 primary tumors had recurrences later than those with T2-4 tumors (28.3 ± 5.5 vs. 12.8 ± 3.0 months, *p* = 0*.*014). Likewise, patients with N0 tumors had recurrences later than those with N1-3 tumors (26.7 ± 4.8 vs. 11.5 ± 3.2 months, *p* = 0*.*013). CD8^+^ TIL and PD-L1 densities in initial tumors had no significant impact on the period to recurrence in this group of patients.

The median value of the CD8 R/I ratio was 0.6 (interquartile range 0.3–2.1). CD8^+^ TIL density showed a decrease (R/I ratio <1) in recurrent tumors compared to initial tumors in 59.5% (25/42) of patients. PD-L1 R/I ratio was also variable, with a median value of 0.4 (interquartile range 0.2–1.2). PD-L1 expression showed a decrease (R/I ratio <1) in recurrent tumors in 69.0% (29/42) of patients. Of note, the expression of PD-L1 in tumor cells showed changes with recurrence in 33.3% (14/42) of patients: from positive to negative in 23.8% of patients and from negative to positive in 9.5% of patients. CD8 R/I ratio was significantly lower in patients with initial stage 4 disease than in those with initial stage 1–3 disease (*p* = 0.010), and in patients who had adjuvant RT than in those who did not have RT (*p* = 0.032). PD-L1 R/I ratio was significantly higher in females than males (*p* = 0.023). Other clinicopathological factors failed to show any association with changes of CD8^+^ TILs or PD-L1 (Table 2).

**Table 2:** Change of CD8^+^ TIL and PDL1 according to the characteristics of initial tumor.

		N	CD8 R/I ratio			PD-L1 R/I ratio		
			Median	1st–3rd quartile	p value*	Median	1st–3rd quartile	p value*
Age	< 60	22	0.42	0.23–1.42	0.087	0.33	0.15–1.25	0.513
	≥ 60	20	1.18	0.39–2.77		0.41	0.17–1.37	
Sex	Female	10	0.27	0.23–0.71	0.098	1.10	0.13–0.96	0.023
	Male	32	0.78	0.35–2.58		0.21	0.33–4.74	
Initial stage	1–3	27	1.19	0.39–2.82	0.010	0.39	0.19–1.49	0.470
	4	15	0.31	0.19–0.58		0.34	0.14–1.03	
Primary site	Oral cavity	21	0.43	0.22–1.98	0.505	0.39	0.20–2.06	0.428
	Others	21	0.65	0.36–2.55		0.34	0.13–1.06	
RT	Not done	12	1.52	0.58–2.48	0.032	1.06	0.21–4.77	0.079
	Done	30	0.41	0.21–1.59		0.33	0.14–0.98	

*R/I ratio* recurrent-to-initial ratio, *SD* standard deviation, *PD-L1* programmed death-ligand 1, *RT* radiation therapy.

*Mann–Whitney’s U test.

**Figure 2:**
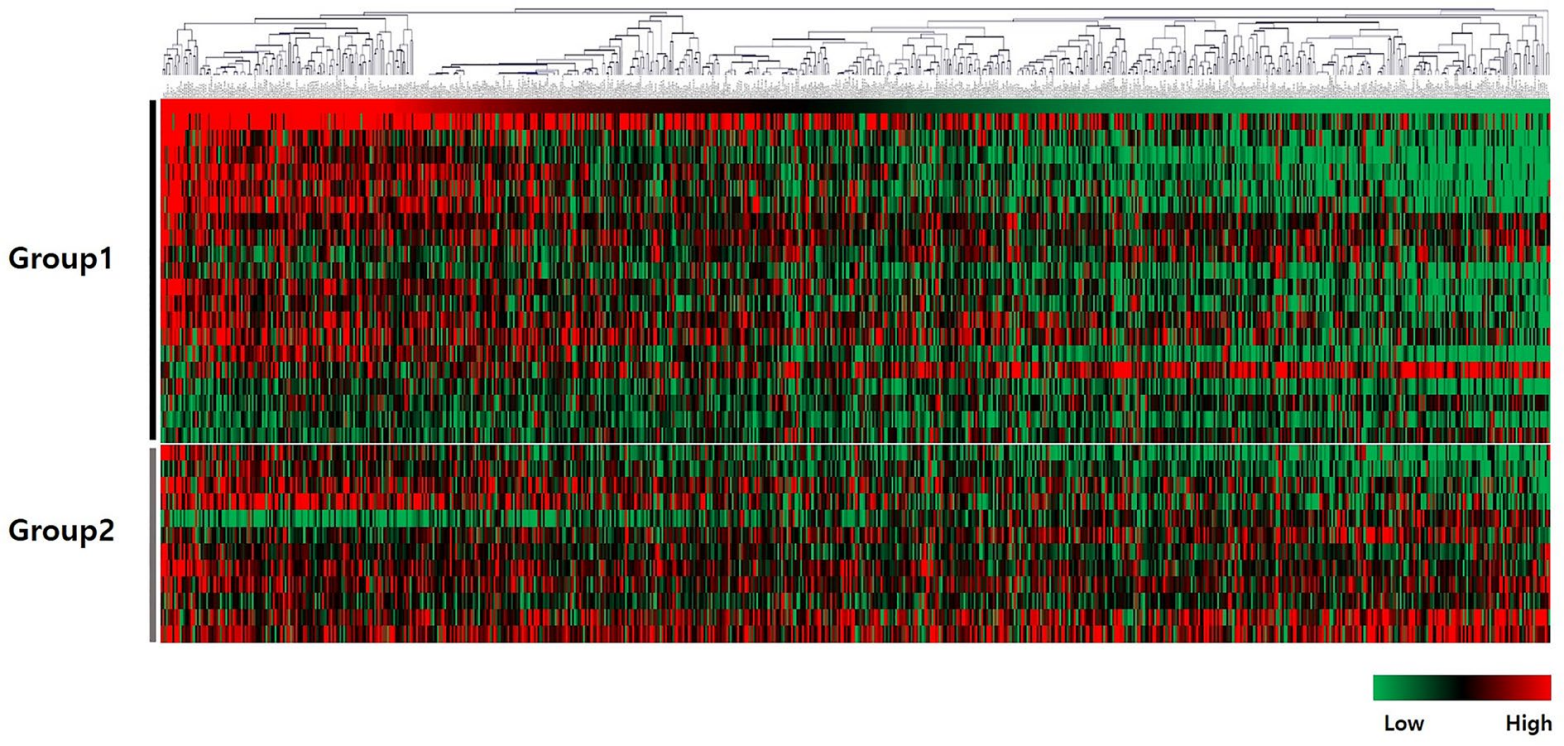
Heatmaps of mRNA expression of 730 immune-related genes in 66 tumor samples from 33 patients. Patients were divided into two groups of patients: those with CD8 R/I < 1 (group1) and those with CD8 R/I > 1 (group2). A heatmap was made using the R/I ratio of each gene. Y-axis was clustered into two groups (groups 1 and 2), and x-axis was clustered hierarchically. A part of the heatmap showed a noticeable pattern between groups 1 and 2.

To validate CD8^+^ TIL changes in IHC staining, relative mRNA expression of each immune-related gene was investigated by mRNA expression profiling. We classified the 33 patients into two groups according to CD8 R/I ratio in IHC: those with CD8 R/I ratio < 1 (group 1) and those with CD8 R/I ratio > 1 (group 2). The R/I ratio of each patient's gene was calculated, and a heatmap was made using the R/I ratios of genes (Fig. 2). The x-axis indicated 730 immune-related genes. The y-axis of the heatmap indicated 33 patients included in two groups. There were noticeable increases in T-cell receptor (TCR)-related genes and T-cell inhibitory genes in group 2 compared to group 1 (Table 3). CD8 antigen encoded by the CD8A gene is a cell surface glycoprotein on most CD8^+^ T lymphocytes. It acts as a co-receptor with TCR. R/I of the CD8A gene was significantly higher in group 2 than in group 1 (median; 1.41 vs. 0.34, *p* = 0*.*008). Likewise, R/I ratios of other TCR-related genes (CD2, CD27, CD3D, CD7, and CD96) were significantly different between the two groups. These TCR-related genes generally showed increases in expression at recurrence (R/I ratio > 1) in group 2, whereas they showed decreases at recurrence (R/I ratio < 1) in group 1. R/I ratios of T-cell inhibitory genes such as CTLA4 and PDCD1 were also higher in group 2 than in group 1.

**Table 3:** Patterns of mRNA expression of immune-related genes according to CD8 R/I ratio measured by immunohistochemical staining.

	CD8 R/I ratio < 1		CD8 R/I ratio > 1		*p-*value*
	n	Median (1st -3rd quartile)	n	Median (1st–3rd quartile)	
**TCR related genes**					
*CD2* R/I ratio	21	0.55 (0.38–0.98)	12	1.10 (0.73–2.60)	0.030
*CD27* R/I ratio	21	0.41 (0.18–0.83)	12	1.46 (0.81–2.51)	0.007
*CD3D* R/I ratio	21	0.50 (0.27–0.74)	12	1.25 (0.76–2.50)	0.004
*CD7* R/I ratio	21	0.58 (0.33–0.95)	12	1.21 (0.57–2.96)	0.018
*CD8A* R/I ratio	21	0.34 (0.26–0.75)	12	1.41 (0.62–1.99)	0.008
*CD96* R/I ratio	21	0.60 (0.39–0.93)	12	1.26 (0.70–2.79)	0.011
**T cell inhibitory genes**					
*CTLA4* R/I ratio	21	0.56 (0.31–0.81)	12	1.23 (0.55–2.70)	0.010
*PDCD1* R/I ratio	21	0.53 (0.29–0.84)	12	1.34 (0.46–2.18)	0.030

*TCR* T-cell receptor, *R/I ratio* recurrent-to-initial ratio, *SD* standard deviation.

*Mann–Whitney’s U test.

Results of association analysis of each factor and OS are presented in Table 4. The mean estimated OS ± standard error was 86.6 ± 13.4 months in patients with a higher CD8^+^ TIL density in the initial tumor and 117.6 ± 22.3 months in those with a lower CD8^+^ TIL density in the initial tumor, showing no significant difference between the two (*p* = 0*.*818). Likewise, patients were divided into two groups according to CD8^+^ TIL density in the recurrent tumor, PD-L1 density in the initial tumor, and PD-L1 density in the recurrent tumor, based on each median value (107.1/μm^2^, 86,576.7/μm^2^, and 50,173.3/μm^2^, respectively). Estimated OS did not differ significantly between recurrent CD8^+^ TILs groups, between initial PD-L1 groups, or between recurrent PD-L1 groups.

**Table 4:** Association analysis between clinico-pathological factors and overall survival.

Factors		n	Estimated survival (*mo*)*	p value^†^
Age	< 60	22	126.2 (20.5)	0.317
	≥ 60	20	74.5 (12.9)	
Sex	Female	10	71.3 (22.1)	0.167
	Male	32	124.4 (17.1)	
Primary site	Oral cavity	21	112.2 (21.5)	0.945
	Others	21	84.1 (12.3)	
Initial Stage	1–3	27	142.2 (18.6)	0.004
	4	15	53.7 (15.2)	
CD8 in initial tumor	< median	21	117.6 (22.3)	0.818
	≥ median	21	86.6 (13.4)	
PD-L1 in initial tumor	< median	21	88.5 (13.2)	0.891
	≥ median	21	117.0 (21.3)	
CD8 in recurrent tumor	< median	21	90.2 (21.4)	0.101
	≥ median	21	105.5 (12.4)	
PD-L1 in recurrent tumor	< median	21	136.1 (20.0)	0.079
	≥ median	21	71.3 (13.9)	
CD8 R/I ratio	< 1	25	65.1 (12.1)	0.003
	> 1	17	167.6 (19.1)	
PD-L1 R/I ratio	< 1	29	124.7 (18.0)	0.661
	> 1	13	86.8 (16.3)	

*NLR* neutrophil-to-lymphocyte ratio, *PLR* platelet-to-lymphocyte ratio, *R/I ratio* recurrent-to-initial ratio, *PD-L1* programmed death-ligand 1.

*presented as a mean (standard error).

^†^Log-rank test.

However, when patients were divided according to CD8 R/I ratio (R/I < 1 vs. R/I > 1), those with increased CD8^+^ TILs at recurrence (CD8 R/I ratio > 1) had significantly better OS (167.6 ± 19.1 months) than those with decreased CD8^+^ TILs at recurrence (R/I ratio < 1) (65.1 ± 12.1 months) (*p* = 0*.*003). PD-L1 R/I ratio had no significant association with OS.

In univariate analysis, stage 4 initial tumor and CD8 R/I ratio were significant factors associated with OS (Table 5). Patients with initial stage 4 tumors had significantly low OS (53.7 ± 15.2 months) than those with initial stage 1–3 tumors (142.2 ± 18.6 months) (*p* = 0*.*004). Age, sex, and primary site had no significant impact on the OS.

**Table 5:** Prognostic factor analysis for overall survival.

		Univariate analysis			Multivariate analysis		
		H.R	95% C.I	*p*-value^†^	H.R	95% C.I	*p*-value^†^
Age	< 60	reference					
	≥ 60	1.570	0.643–3.835	0.322			
Initial stage	1–3	reference			reference		
	4	3.371	1.387–8.189	0.007	2.236	0.869–5.753	0.095
CD8 R/I ratio	< 1	reference			reference		
	> 1	0.218	0.072–0.660	0.007	0.293	0.091–0.945	0.040
PD-L1 R/I ratio	< 1	reference					
	> 1	1.229	0.487–3.099	0.663			

*R/I ratio* recurrent-to-initial ratio, *PD-L1* programmed death-ligand 1, *H.R.* Hazard ratio, *C.I.* confidence interval, *R/I ratio* recurrent-to-initial ratio.

*Mean (standard error), ^†^Cox regression model.

A Cox regression model indicated that CD8 R/I ratio was the only good prognostic factor in patients with local or locoregional recurrence after surgery-based treatment for HNSCCs (HR: 0.293, 95% CI: 0.091–0.945, *p* = 0*.*040). Initial stage 4 tumors did not reach statistical significance.

**Figure 3:**
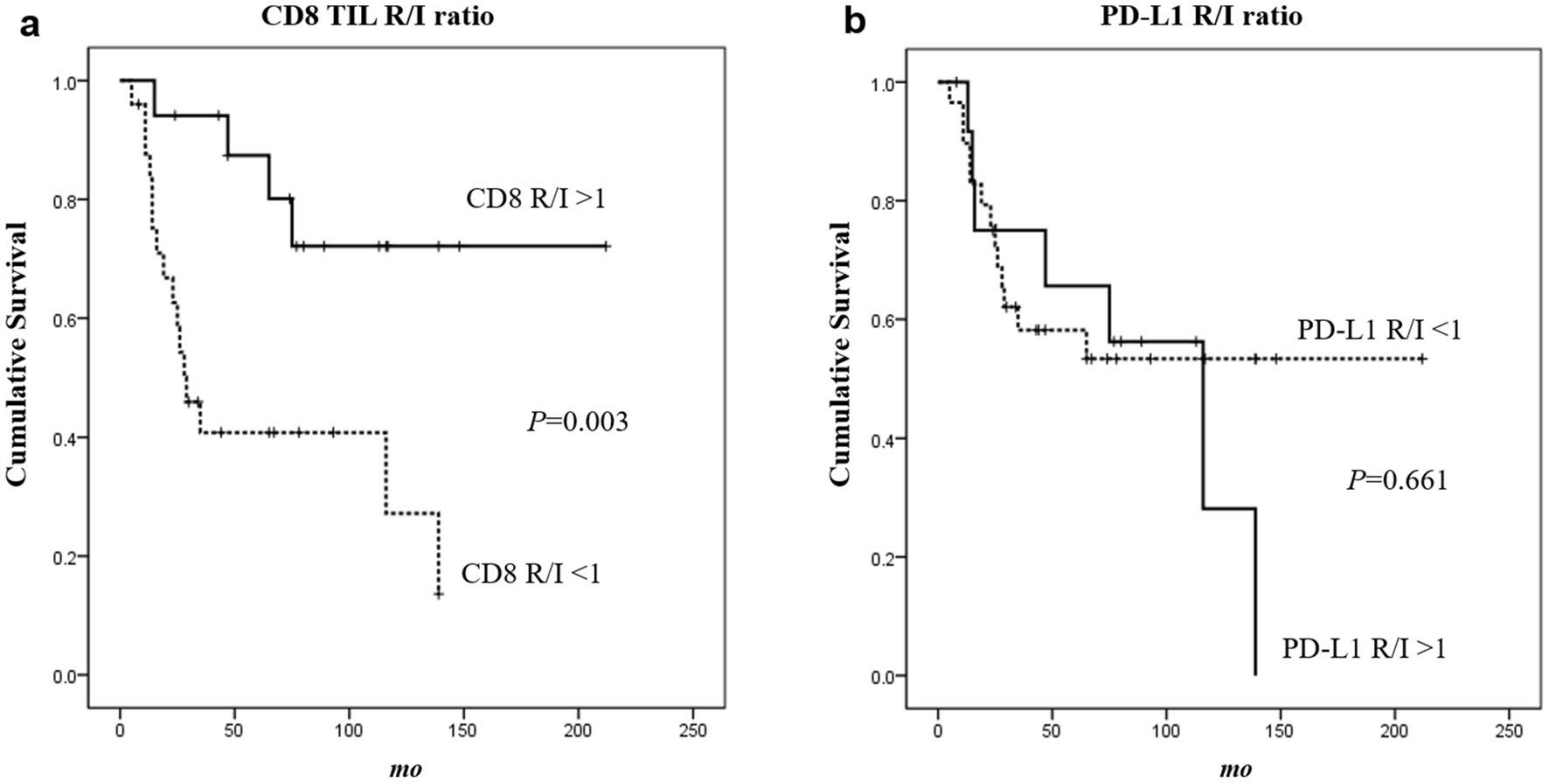
Overall survival (OS) plots according to CD8^+^ tumor-infiltrating lymphocytes (TILs) and PD-L1 expression. (**a**) Patients with increased CD8^+^ TILs at recurrence (R/I ratio > 1) had significantly better OS (167.6 ± 19.1 months) than those with decreased CD8^+^ TILs (R/I ratio < 1) (65.1 ± 12.1 months) (*p* = 0*.*003). (**b**) PD-L1 R/I ratio had no significant association with OS.

By Kaplan-Meir estimates, patients with increased CD8^+^ TILs at recurrence (CD8 R/I ratio > 1) had significantly better OS (167.6 ± 19.1 months) than those with decreased CD8^+^ TILs at recurrence (R/I ratio < 1) (65.1 ± 12.1 months) (Log rank *p* = 0*.*003) (Fig. 3a). PD-L1 R/I ratio had no significant association with OS (Fig. 3b).

## Discussion

Biomarkers can be used to select appropriate patients for anti-PD1/PD-L1 immunotherapy and to predict treatment outcome. Given that overall response rates to anti-PD1/PD-L1 immunotherapy were only 13.3–17.7% in recurrent and metastatic HNSCCs in previous clinical trials, identifying predictive biomarkers is essential^5–7^. TIL infiltration and PD-L1 expression are known as the two most proficient biomarkers for anti-PD1/PD-L1 immunotherapy. PD-L1 expression assessed by IHC is the most common clinically used and officially approved biomarker for PD-1/PD-L1 blockade. It has been reported that patients with PD-L1 expression have better responses to anti-PD-1/PD-L1 immunotherapy^5–7,19,20^. The infiltration of TILs, more specifically, CD8^+^ TILs, has also been mentioned as a critical factor. It has been reported that patients with both intense TIL infiltration and PD-L1 expression are the ones who are most likely to benefit from anti-PD1/PD-L1 immunotherapy^9,20^. Furthermore, some studies have concluded that TIL infiltration, not PD-L1 expression, is a critical factor in determining cancer immunotherapy responsiveness. Tumeh et al. have reported that invasive margin CD8^+^ TIL density is a better predictor of response to a PD-1 inhibitor (pembrolizumab) over PD-1 or PD-L1 expression^11^.

In this study, we found that the increase of CD8^+^ TILs in the recurrent tumor was a significant prognosticator for OS in HNSCC patients with local recurrence. Changes in PD-L1 expression failed to show any impact on OS in this group of patients. This finding is interesting because the change in CD8^+^ TILs has not been studied concerning the prognosis. The prognostic value of CD8^+^ TILs in initial HNSCCs has been demonstrated well in previous studies, including meta-analysis^21–23^. However, in this study, initial CD8^+^ TIL density itself did not have a significant association with OS. This difference in prognostic significance of CD8^+^ TILs may have been attributed to the difference in study populations. While the prognostic role of CD8^+^ TILs had been previously studied in the overall patients with HNSCC, this study only included the patients with recurrence. Within population with recurrent HNSCC, the prognostic value of CD8^+^ TILs for OS can be different from that in overall population with HNSCC. Instead, for the patients' group included in this study, an increase of CD8^+^ TILs in recurrent tumors (R/I ratio > 1) had an HR of 0.293 (95% CI: 0.091–0.945) for OS. The final OS was better in patients with increased CD8^+^ TILs at recurrence. The bottom-line finding of the present study was that the change, not the one-time value of CD8^+^ TILs, affected the survival of patients with local recurrence.

CD8^+^TIL changes observed with IHC staining were validated with mRNA expression profiling in this study. In patients with increased CD8^+^ TILs at recurrence, expression of TCR-related genes (CD8A, CD2, CD27, CD3D, CD7, and CD96) generally increased at recurrence. These genes are involved in the activation and maintenance of T-cell reactions. Interestingly, expression levels of CTLA4 and PDCD1 genes also increased in patients with increased CD8^+^ TILs at recurrence. CTLA4 and PDCD1 genes are two representative immune checkpoint genes. At recurrence, these inhibitory genes' increases can be triggered at the early and late phases of T-cell reaction. Thus, they can be T-cell exhaustion markers. If accompanying increases in the expression of these inhibitory genes or proteins were blocked in patients with increased CD8^+^ TILs at recurrence, their oncological outcome could be improved.

The prognostic implication of PD-L1 is currently unclear. High expression of PD-L1 in tumors is considered a poor prognostic factor because PD-L1 suppresses T-cell reaction against tumors^24,25^. However, the up-regulation of PD-L1 expression could result from a vigorous immune response against tumors^25^. To date, there have been conflicting results on the prognostic implication of PD-L1 expression^26,27^. The prognostic role of PD-L1 expression differs widely between studies. Meta-analyses have failed to prove a significant correlation between PD-L1 expression and survival endpoints in HNSCC patients^28,29^. In the present study, the change of PD-L1 at recurrence (PD-L1 R/I ratio), as well as PD-L1 densities in initial and recurrent tumors, also failed to benefit OS significantly (*p* = 0*.*661), which is not a surprising result considering previous studies. The small number of included patients and/or specific inclusion criteria (only patients with local/locoregional recurrence after surgery-based treatment included) might have limited the statistical significance. It would be more reasonable to evaluate the prognostic implication of PD-L1 expression in combination with the status of other immunologic profiles. Also, in vitro evidence shows that PD-L1 expression can be altered by various agents such as cisplatin^14,15^. Ock et al. have reported that PD-L1 expression changed during cisplatin treatment in patients with HNSCCs^13^. In their report, the status of PD-L1 expression changed after cisplatin treatment in 37.1% (13/35) cases (positive conversion in nine cases and negative conversion in four cases). Although one study has shown that PD-L1 expression level is high in both recurrent and metastatic oropharyngeal carcinomas (43% and 70%, respectively), there has been no report on changing immunologic profiles according to recurrence in HNSCC^30^. Also, the prognostic impact of such a change has not been reported yet.

The prognostic role of PD-L1 can also be different according to the cells in which it is expressed. Kim et al. have reported that PD-L1 expression is high on immune cells but not on tumor cells^31^. They also reported that a high expression level of PD-L1 was an independent favorable prognostic factor for RFS and OS^31^. In the case of high PD-L1 cases in this study, PD-L1 expression is frequently observed in tumor cells. In low PD-L1 cases, there is a tendency for PD-L1 to be expressed primarily in stromal lymphocytes (Supplementary Fig. 1). However, we evaluated the overall density of PD-L1, including immune cells, macrophages, and tumor cells for analysis. This overall density is somewhat like a 'combined positive score,' a term used in many previous studies (the number of PD-L1^+^ cells divided by the number of tumor cells). This could be a limitation of this study. If we could analyze PD-L1 density on TILs separately, more detailed information on the prognostic implication of change in PD-L1 expression could have been obtained.

This study has several limitations, such as a small number of patients, retrospective design, and different cancer treatment characteristics between patients. However, this study's methodology, a paired analysis of initial and recurrent tumors, are rare and worth noting. According to the present study, in patients who had recurrence after initial treatment for HNSCCs, changes of CD8^+^ TILs and PD-L1 were widely variable. The change of CD8^+^ TILs at recurrence was accompanied by mRNA changes in TCR-related genes and T-cell inhibitory genes. In the group of patients with recurrence after initial surgery-based treatment for HNSCCs, increased CD8^+^ TILs at recurrence was an excellent independent prognosticator, which can be useful information in immune checkpoint inhibitor therapy.
